# Predicting Athletes’ Pre-Exercise Fluid Intake: A Theoretical Integration Approach

**DOI:** 10.3390/nu10050646

**Published:** 2018-05-21

**Authors:** Chunxiao Li, Feng-Hua Sun, Liancheng Zhang, Derwin King Chung Chan

**Affiliations:** 1 Department of Health and Physical Education, The Education University of Hong Kong, Hong Kong, China; fhsun@eduhk.hk; 2 Key Laboratory of Competitive Sport Psychological and Physiological Regulation, Tianjin University of Sport, Tianjin 301617, China; zlc-hhht@163.com; 3 School of Public Health, Li Ka Shing Faculty of Medicine, The University of Hong Kong, Hong Kong, China; derwin.chan@hku.hk

**Keywords:** self-determination, planned behavior, intention, beverage consumption, sport

## Abstract

Pre-exercise fluid intake is an important healthy behavior for maintaining athletes’ sports performances and health. However, athletes’ behavioral adherence to fluid intake and its underlying psychological mechanisms have not been investigated. This prospective study aimed to use a health psychology model that integrates the self-determination theory and the theory of planned behavior for understanding pre-exercise fluid intake among athletes. Participants (*n* = 179) were athletes from college sport teams who completed surveys at two time points. Baseline (Time 1) assessment comprised psychological variables of the integrated model (i.e., autonomous and controlled motivation, attitude, subjective norm, perceived behavioral control, and intention) and fluid intake (i.e., behavior) was measured prospectively at one month (Time 2). Path analysis showed that the positive association between autonomous motivation and intention was mediated by subjective norm and perceived behavioral control. Controlled motivation positively predicted the subjective norm. Intentions positively predicted pre-exercise fluid intake behavior. Overall, the pattern of results was generally consistent with the integrated model, and it was suggested that athletes’ pre-exercise fluid intake behaviors were associated with the motivational and social cognitive factors of the model. The research findings could be informative for coaches and sport scientists to promote athletes’ pre-exercise fluid intake behaviors.

## 1. Introduction

Exercise is defined as a planned, structured, and repetitive physical activity for improving or maintaining physical fitness [1]. To start the exercise with a normal state of body water content, athletes should drink enough fluid or should be well hydrated prior to exercise [2]. The goal of pre-exercise fluid intake is critical for decreasing the risk of dehydration (loss of body water) and its negative health consequences (e.g., heart disease), and maintaining exercise performance [3]. Sawka et al. [3] proposed an evidence-based guideline of pre-exercise fluid intake. In particular, athletes are advised to slowly intake fluid (e.g., 5–7 mL/kg per body weight) at least four hours prior to exercise, and the fluid intake should be increased (e.g., 3–5 mL/kg per body weight) and be taken about two hours before exercise when athletes are dehydrated (i.e., dehydration is indicated by having no urine or highly concentrated urine). Many athletes seem to not be committed to this recommended behavior [4]. Although pre-exercise fluid intake could be facilitated by increasing drinking-water facilities and improving the convenience of executing the behavior, the actual pre-exercise fluid intake is also highly dependent on decision making factors, motivation, and commitment [5,6].

It is, therefore, valuable to understand the underlying psychological mechanisms of pre-exercise fluid intake and the results might be important to explain why some athletes are not committed to this advisory behavior. The present study aims to apply a unified health psychology model that integrates self-determination theory (SDT) [7] and the theory of planned behavior (TPB) [8] in order to understand athletes’ pre-exercise fluid intake.

It is conceptualized in the integrated model that human health behaviors are governed by distal motivational factors from SDT and proximal decision-making factors from TPB [9]. According to SDT, there are two broad forms of motivation behind human actions, including autonomous motivation and controlled motivation [10]. *Autonomous motivation* reflects those motivational behaviors that are consistent with a sense of volition and choice. In contrast, *controlled motivation* is concerned with those motivational behaviors that are regulated by external contingencies, such as rewards/punishment and internal pressure to avoid feelings of guilt and shame [10]. As compared to controlled motivation, autonomous motivation is considered to be more adaptive [10], and it is more likely to lead to behavioral persistence and psychological well-being. However, it is postulated in the integrated model that the relationship between motivations and behavior is not direct, but it is mediated by the decision-making factors from the TPB [9].

The TPB is a social-cognitive model [8], in which the intention is regarded as the central predictor of one’s behavior (e.g., fluid intake behavior). According to the TPB [8], three sets of social cognitive variables (i.e., attitude, subjective norm, and perceived behavioral control [PBC]) positively predict behavior via intention. PBC is also proposed to directly predict behavior. *Attitude* concerns one’s overall subjective evaluation towards the target behavior. *Subjective norm* reflects one’s perception of how the behavior is regarded as being socially appropriate. *PBC* summarizes one’s personal judgement on capacity of engaging in the target behavior [8]. 

The theoretical components of SDT and TPB are merged into the integrated model, such that autonomous motivation (rather than controlled motivation) from SDT is speculated to positively predict attitude, subjective norm, and PBC from the TPB. These three social-cognitive variables further link intention and behavior according to the tenets of the TPB [9]. Integrating these two theoretical frameworks may bring forth a more comprehensive understanding towards health behaviors because the theoretical integration combines the merits and it resolves the limitations of the theories. Specifically, SDT supplements the TPB by providing the superordinate motivational antecedents that account for the origin of the social cognitive process. On the other hand, the TPB accounts for the proximal belief-oriented decision-making process [9].

The integration of SDT and the TPB has been successfully applied in fields, such as anti-doping [11], physical activity [12], prevention and rehabilitation of injury [13], myopia prevention [14], and sleep hygiene [15]. Early studies that are guided by the integrated framework generally indicated that autonomous motivation positively predicted attitude, subjective norm, and PBC, while controlled motivation typically exerted either small or no effects on these three belief-based TPB constructs [12,14]. More recent research findings began to reveal a positive relationship between controlled motivation and subjective norms [11,13]. This is because social approval, external pressure, and recognition of behavior could be regarded as externally referenced motives, and they may be also closely linked to individuals’ normative beliefs [11,13]. In addition, the three TPB constructs had a positive effect on healthy behaviors through intention [9,14,15]. Although the utility of the integrated framework in explaining fluid intake behaviors has not been explored, studies applying either SDT or the TPB alone have been conducted in the context of healthy diets. Findings of these early studies could somewhat provide information about the potential application of the integrated model into fluid intake behavior [16,17].

For instance, a few empirical studies that are based on SDT found that autonomous motivation promoted adoption of healthy eating behaviors and controlled motivation showed no or little effect on healthy diets [16,18]. A recent meta-analysis summarizing the findings of 34 studies about the TPB and dietary behaviors among youth showed that the relationship between the three social cognitive factors, intention, and dietary behavior agreed with the tenets of the TPB [17]. Although these studies only focused on a healthy diet, given pre-exercise fluid intake is also a self-regulatory dietary behavior specifically for athletes and physically active individuals, the findings of SDT and the TPB on healthy diets lead to speculation that the integration of both the theories could be useful in explaining athletes’ pre-exercise fluid intake behavior.

**Figure 1:**
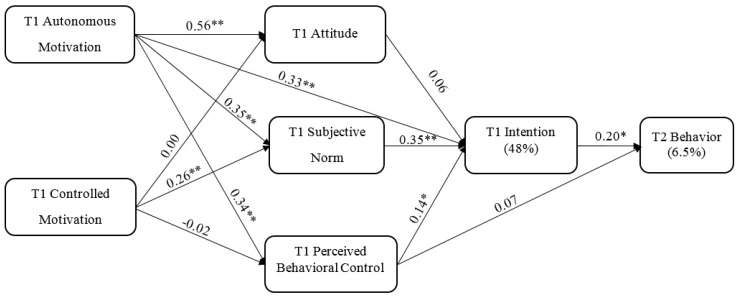
Path estimates of the model. T1 = baseline; T2 = one-month after the baseline; * *p* < 0.05; ** *p* < 0.01. For clarity, correlations between gender and major study variables are omitted.

Although there is an evidence-based guideline for pre-exercise fluid intake, little is known about the underlying psychological mechanisms for this healthy behavior among athletes. From a practical perspective, understanding the underlying mechanisms will help practitioners (e.g., coaches and trainers) and researchers to establish evidence-based intervention programs to promote healthy pre-exercise fluid intake behaviors. Therefore, this two-wave prospective research was undertaken in order to test the utility of the integrated framework consisting of SDT and the TPB in predicting pre-exercise fluid intake among university athletes (see Figure 1). According to our literature review above [8,9,17], it was hypothesized that:

(H1) Autonomous motivation would be a positive predictor of the three social cognitive variables (i.e., attitude, subjective norm, and PBC).

(H2) Controlled motivation would be positively related to subjective norm, but its effect on attitude and PBC would be either small or insignificant.

(H3) Autonomous motivation and the three social cognitive variables were expected to positively predict intention.

(H4) Intention would positively predict pre-exercise fluid intake behavior.

(H5) The three social cognitive variables were proposed to mediate the predictive effects of autonomous/controlled motivation on intention. 

(H6) Intention was expected to mediate the relationship between the three social cognitive variables and pre-exercise fluid intake behavior (i.e., full mediation for attitude, subjective norm, and partial mediation for PBC).

(H7) Autonomous/controlled motivation would have an indirect effect on the fluid intake behavior via the three social cognitive variables and intention.

## 2. Materials and Methods

A two-wave prospective survey design with a one-month interval between the baseline assessment and the prospective follow-up was used in the current research.

### 2.1. Participants

A sample of university athletes (*n* = 182) was recruited from three public universities in China. Participants had a mean age of 20.75 (*SD* = 2.24) and 66.5% of them were male. The participants were from a wide range of sports (*n* = 13), such as badminton, soccer, and volleyball. On average, they received training for 5.51 (*SD* = 3.40) years and trained 9.28 h per week (*SD* = 5.39).

### 2.2. Measures

#### 2.2.1. Autonomous and Controlled Motivation

We adapted 12 items from the Chinese version of the Treatment Self-Regulation Questionnaire for assessing the autonomous and controlled motivation (see Table S1) [13]. The scale has been used for measuring motivation of the integrated model in various behavioral contexts that are related to the prevention or management of injury or illnesses [11,14]. There are six items for evaluating each broad form of motivation including autonomous motivation (e.g., “I want to intake sufficient fluid before exercise because it is consistent with my life goals”) and controlled motivation (e.g., “I want to intake sufficient fluid before exercise because I would feel ashamed of myself if I did not”). Participants gave responses on seven-point Likert scales, ranging from “*not at all true*” (1) to “*very true*” (7).

#### 2.2.2. Social Cognitive Constructs and Intention

Items measuring attitude, subjective norm, PBC, and intention were again adapted from the Chinese version of the TPB scale that was developed and used in prior studies of the integrated model conducted in China (see Table S1) [11,14]. The common stem (“Intake sufficient fluid before exercise is…”) was employed to assess participants’ attitude, and they made responses on five seven-point semantic differential scales, which included “valuable–worthless”, “beneficial–harmful”, “pleasant–unpleasant”, “enjoyable–unenjoyable”, and “good–bad”. Measures of subjective norm (three items; e.g., “The people in my life whose opinions I value would approve of me consuming sufficient fluid before exercise in the forthcoming month”), PBC (four items; e.g., “I have complete control over consuming sufficient fluid before exercise in the forthcoming month”), and intention (three items; e.g., “I intend to consume sufficient fluid before exercise in the forthcoming month”) were rated on seven-point Likert scales that were anchored from “*strongly disagree*” (1) to “*strongly agree*” (7).

#### 2.2.3. Pre-Exercise Fluid Intake Behavior

Since the required amount of pre-exercise fluid intake is determined by a number of factors (e.g., the type of fluid, individual differences, sport type, temperature, and humidity) [3], measuring the exact amount of pre-exercise fluid intake might not necessarily indicate the hydration status of the athletes. The study, therefore, aims to examine the behavioral adherence that athletes apply toward maintaining an optimal hydration status before exercise, and it requires a self-regulatory effort in monitoring the hydration status and intake fluid when necessary (e.g., monitor one’s weight). With the input from one sport physiologist and two sport psychologists, four items (e.g., “I will observe my urine color three to four hours prior to exercise”) were developed to measure behavioral adherence to pre-exercise fluid intake, according to the guidelines of Sawka et al. (see Table S1) [3]. Pilot testing with 10 athletes indicated that no item revisions were necessary. Participants rated on the items using five-point Likert scales, which ranged from “*almost never*” (1) and “*almost always*” (5). Since this is a newly developed scale, we conducted an exploratory factor analysis to examine its underlying structure. The results supported the one-factor model of the scale (Eigenvalue = 2.14), and the four items explained 53.6% of the total variance in pre-exercise fluid intake.

### 2.3. Procedures

The Human Research Ethics Committee of The Education University of Hong Kong granted ethical approval of this research on 20 May 2016 (ref. No. 15216). The first author contacted course lecturers from three public universities located in Southeastern and Northern China to invite the university team athletes to participate in this research. The course lecturers then invited their athletes to participate in this survey through a social media platform. Upon obtaining participants’ informed consent, the survey form was distributed to them during their scheduled lecture hours in quiet classrooms (Time 1). A follow-up behavioral measure (i.e., fluid intake behavior) was taken one month later (Time 2) in the same class. The one-month interval between the two measurement points has been recommended [19]. For both administration occasions, participants were under supervision of researchers and were encouraged to provide honest responses. Special emphasis was placed on confidentiality and no mandatory participation. Of 200 athletes invited, 182 (91%) agreed to participate and filled out the survey form at Time 1 and 179 completed the survey at Time 2 (response rate at follow-up = 98.4%).

### 2.4. Data Analyses

Since there was only a small amount of missing values (0.4%) among study variables, the missing values were imputed through variable means in SPSS 21 (IBM, Armonk, NY, USA) [20]. Cronbach’s alphas (*α*), means, standard deviations, and zero-order correlations of the study variables were computed. A path analysis with a maximum likelihood estimation was applied to test the overall fit of the hypothesized model and hypotheses in AMOS 21 (IBM, Armonk, NY, USA) [21]. Gender was entered as a co-variate since it was found to affect the strength of path estimates [9]. The current sample size (*n* = 179) is generally adequate for path analysis [20]. Model fit was evaluated through chi-square statistics to the degree of freedom ratio (*χ*²/*df*), comparative fit index (CFI), Tucker-Lewis index (TLI), root mean square error of approximation (RMSEA), and standardized root mean square residual (SRMR) [22]. A value of *χ*^2^/*df* smaller than 3.0, CFI/TLI values over 0.95, and RMSEA/SRMR values that are less than 0.06 represent a good fit [23]. To examine the mediation effects, mediation analyses using bootstrapping approach with 5000 replications were used to generate bias-corrected confidence intervals (CIs) of path estimate. A 95% CI that did not include zero indicates a significantly indirect effect [24].

## 3. Results

Table 1 presents the results of internal reliability, descriptive statistics, and zero-order correlations. The (sub) scales showed an adequate to excellent internal reliability (Cronbach’s *α* = 0.71 to 0.92). Participants reported moderate to high levels of autonomous/controlled motivation, attitude, subjective norm, PBC, behavioral intention, and fluid intake behavior. The zero-order correlations between the study variables yielded small to medium effect sizes.

The results of the path analysis supported the proposed model: *χ*²_(5)_ = 6.90, *χ*²/*df* = 1.38, CFI = 0.995, TLI = 0.971, RMSEA = 0.046, SRMR = 0.030. Figure 1 presents the detailed path estimates. In line with H1 and H2, autonomous motivation positively predicted the three social cognitive variables (*β* = 0.34 to 0.56, *p* < 0.01), while controlled motivation exerted no effects on attitude/PBC (*β* = 0.00/−0.02, *p* = 0.98/0.85), but a significant effect on subjective norms (*β* = 0.26, *p* < 0.01). Autonomous motivation (*β* = 0.33, *p* < 0.01), subjective norm (*β* = 0.35, *p* < 0.01), and PBC (*β* = 0.14, *p* = 0.03) were positive predictors of intention, which subsequently predicted pre-exercise fluid intake behavior (*β* = 0.23, *p* < 0.01). Therefore, H3 was partially supported and H4 was confirmed. Autonomous/controlled motivation and the three social cognitive variables explained 48% of the total variance in intention. SDT- and TPB-based constructs only explained 6.5% of the total variance in behavior.

Table 2 shows the results of mediation analysis. In line with H5, the three social cognitive variables were found to mediate the relationships between autonomous/controlled motivation and intention (*β* = 0.20/0.09, *p* = 0.00/0.02). The direct link between PBC and pre-exercise fluid intake was not significant (*β* = 0.07, *p* = 0.39). Furthermore, the relationships between subjective norm/PBC and pre-exercise fluid intake behavior were fully mediated by intention (*β* = 0.07/0.03, *p* = 0.01/0.03), and intention was not a mediator in the path between attitude and behavior (*β* = 0.01, *p* = 0.36). Therefore, H6 was partially supported. H7 was partially confirmed given autonomous motivation (*β* = 0.13, *p* = 0.003), but not for controlled motivation (*β* = 0.02, *p* = 0.23) had an indirect effect on behavior via the three social cognitive variables and intention.

## 4. Discussion

The aim of the present prospective study was to use a multi-theory model that integrates SDT- and TPB-based constructs and hypotheses to understand athletes’ pre-exercise fluid intake. Findings generally supported a number of effects found in early research that applied the integrated model to understand other health behaviors [9,14,15], including the effects of autonomous motivation and the three social cognitive variables on intention, which subsequently predicted pre-exercise fluid intake behavior. Overall, SDT- and TPB-based variables explained a small amount of the total variance in pre-exercise fluid intake behavior (6.5%).

Focusing first on the proposed prediction of the three social cognitive variables by autonomous motivation (H1), our results are parallel to earlier research that showed that autonomous motivation was a positive predictor of the three social cognitive variables [9,12,14]. This finding implies that the more the athletes were autonomously motivated to engage in taking fluid before exercise, the more likely they were to hold a positive evaluation of the behavior, regarding such behavior as socially appropriate, and perceive a strong sense of personal control over the action. H2 was also supported in that controlled motivation was a positive predictor of subjective norms, but not for attitude and PBC after controlling for autonomous motivation. Previous research has shown that controlled motivation relative to autonomous motivation has a limited role in predicting the three social cognitive variables [16,25]. In addition, since controlled motivation reflects externally referenced contingencies such as obtaining rewards and avoiding social pressure, it tends not to be aligned with attitude and PBC, but with subjective norm, which reflects one’s beliefs about socially appropriate behaviors [25]. Hagger and Chatzisarantis [9] asserted that subjective norms may reflect both autonomous and controlled aspects of social cognitive belief on future health behavior engagement, which explains why both autonomous and controlled motivation were positively related to subjective norm in this research. 

H3, being partially supported as the path from attitude to intention, was not significant. The finding seemed to contradict with the tenets of TPB [8]. However, there was a positive association between attitude and intention (*r* = 0.47, *p* < 0.01), based on the results of zero-order correlation. Therefore, it is highly possible that attitude had a limited effect on intention after controlling for the subjective norm and PBC. Furthermore, the subjective norm exerted a larger effect than PBC on intention (0.35 vs. 0.14). The context where a behavior is situated may affect the predictive ability of the three social cognitive variables [8]. As such, the subjective norm is likely the strongest predictor of intention in the context of pre-exercise fluid intake. There is a strong social component in pre-exercise fluid intake behavior, and university athletes’ intention is mainly influenced by their beliefs about significant others’ expectations (e.g., teammates). Observably, athletes have a lot of exposure to teammates’ normative influences since they train together most of the time.

It is important to note that this is the first research to extend the TPB in understanding athletes’ pre-exercise fluid intake behaviors. Their intention was found to positively predict pre-exercise fluid intake in our study. This finding is consistent with H4 and the TPB [8]. However, the effect size of the intention-behavior link is small (*β* = 0.20) and a similar effect was also found in early prospective research [9,26]. The small effect may be due to the intention-behavior gap [27]. To bridge the intention-behavior gap, theorists have suggested a number of solutions such as including implementation intention, planning, maintenance self-efficacy, action control, and self-control in the process of the intention-behavior relationship [6,26,27,28]. Another possible avenue is to use the dual-process approach [29,30,31]. Some health behaviors may be affected by the impulsive process, in which automaticity or habit implicitly governs the behavioral execution. Future research may incorporate automatic factors (e.g., implicit attitude or habit) into the integrated model that may provide additional avenues for the target behavior [5,25].

With regard to the mediation effects, the relationship between autonomous motivation and intention was partially mediated through the three social cognitive variables, while controlled motivation was fully mediated through them. This finding is consistent with H5 and early research [9,25], which suggests two possible routes by which superordinate motivational antecedents from SDT may predict intention. Namely, a mediated route that includes intention and its proximal predictors (i.e., the three social cognitive variables), and a direct route that directly affects intention independent of the belief-orientated decision-making process [26]. H6 was only partially supported, since intention was found to fully rather than partially mediate the relationship between PBC and fluid intake behavior. Lack of a direct effect from PBC to behavior is possible due to the measure of PBC. Our PBC measure reflects perceived rather than actual control beliefs. According to Ajzen [8], when the PBC measure reflects actual control over behavior, PBC will predict behavior directly. Although we found that autonomous motivation had an indirect effect on fluid intake behavior via the three social cognitive variables and intention, the finding is similar to the findings of meta-analytic studies of the integrated model [9,32]. Although motivation may also form a direct effect on health behavior [25], the mediation of social cognitive variables addresses the importance of understanding the proximal decision-making process to enhance the variance of intention and behavior.

Even though the present research has several strengths (e.g., the first research to test the integrated model in the area of pre-exercise fluid intake behavior), it is not without its limitations. First, the participants of this study were university athletes in China, which means the present findings may not be generalized into athletes in other age groups, educational levels, or cultures. Future research should examine whether the findings can be replicated across diverse populations. Second, the ongoing debate on the effectiveness of pre-exercise hydration status (e.g., [33]) could somewhat reduce the practical implications of the present study. However, the current guidelines are widely accepted, and the effective pre-exercise hydration status has not been proven to be deleterious on performance. Third, we relied entirely on self-reported measures that are subject to recall bias and response tendency [34]. Objective measures representing the effectiveness of behavioral adherence to pre-exercise fluid intake (e.g., urine specific gravity and body weight) should be used in future research. Lastly, even though a two-wave prospective survey design was used in this research, it is insufficient to conclude the temporal and causal nature of the relationships that are found in the integrated model. A panel design or intervention research is necessary to provide stronger evidence for the conclusions.

## 5. Conclusions

Our survey extended and tested the integrated model in the context of pre-exercise fluid intake among athletes. We generally found support for the model and its related hypotheses. Our findings suggest that there are multiple potential routes for pre-exercise fluid intake behavior, which provide preliminary evidence for the development of pre-exercise fluid intake interventions. Intervention programs should target all of the relevant routes to increase the intention and behavior of pre-exercise fluid intake. For example, since autonomous motivation has both substantially direct and indirect effects on intention, it would be useful to increase athletes’ autonomous motivation. According to SDT [10], autonomy-supportive behaviors (e.g., providing choices in selecting different types of fluid and creating a caring climate during behavior changing processes) can be an effective way to enhance their autonomous motivation.

## Figures and Tables

**Table 1:** Descriptive statistics, internal reliability, and zero-order correlations among the study variables.

	1.	2.	3.	4.	5.	6.	7.	8.
1. T1 Autonomous motivation	1							
2. T1 Controlled motivation	0.45 **	1						
3. T1 Attitude	0.56 **	0.24 **	1					
4. T1 Subjective norm	0.47 **	0.40 **	0.49 **	1				
5. T1 Perceived behavior control	0.33 **	0.12	0.43 **	0.47 **	1			
6. T1 Intention	0.58 **	0.36 **	0.47 **	0.59 **	0.43 **	1		
7. T2 Behavior	0.25 **	0.22 **	0.16 *	0.26 **	0.17 *	0.20 **	1	
8. Age	0.10	0.00	0.11	0.05	−0.06	0.09	0.13	1
9. Gender	−0.02	−0.10	0.02	0.19 **	0.15 *	0.07	−0.09	0.03
Range	1–7	1–7	1–7	1–7	1–7	1–7	1–5	17–26
Mean	4.77	2.43	6.02	4.23	5.37	4.73	2.43	20.75
Standard deviation	1.17	1.12	1.15	1.34	1.17	1.49	0.77	2.24
Cronbach’s α	0.78	0.77	0.92	0.75	0.82	0.85	0.71	—

T1 = baseline, T2 = one-month after the baseline, * *p* < 0.05, ** *p* < 0.01.

**Table 2:** Mediation analyses showing the standardized direct, indirect, and the total effects of the model.

Effect (Corresponding Hypothesis If Applicable)	*β*	*p*	95% CI
*Direct effects*			
Autonomous motivation → Attitude (H1)	0.56	<0.001	[0.43, 0.69]
Autonomous motivation → Subjective norm (H1)	0.35	0.001	[0.20, 0.49]
Autonomous motivation → PBC (H1)	0.33	<0.001	[0.17, 0.49]
Controlled motivation → Attitude (H2)	0.00	0.99	[−0.13, 0.13]
Controlled motivation → Subjective norm (H2)	0.26	0.001	[0.11, 0.40]
Controlled motivation → PBC (H3)	−0.02	0.83	[−0.15, 0.12]
Autonomous motivation → Intention (H3)	0.33	<0.001	[0.18, 0.49]
Attitude → Intention (H3)	0.06	0.46	[−0.09, 0.19]
Subjective norm → Intention (H3)	0.35	0.001	[0.17, 0.51]
PBC → Intention (H3)	0.15	0.04	[0.01, 0.27]
Intention → FIB (H4)	0.20	0.01	[0.04, 0.35]
*Indirect effects*			
Autonomous motivation → TPB constructs → Intention (H5)	0.20	<0.001	[0.10, 0.32]
Controlled motivation → TPB constructs → Intention (H5)	0.09	0.02	[0.02, 0.18]
Attitude → Intention → FIB (H6)	0.01	0.36	[−0.02, 0.05]
Subjective norm → Intention → FIB (H6)	0.07	0.01	[0.01, 0.15]
PBC → Intention → FIB (H6)	0.03	0.03	[0.01, 0.08]
Autonomous motivation → TPB constructs → Intention → FIB (H7)	0.13	0.003	[0.04, 0.22]
Controlled motivation → TPB constructs → Intention → FIB (H7)	0.02	0.23	[−0.01, 0.05]
*Total effects*			
Autonomous motivation → Intention	0.54	<0.001	[0.42, 0.64]
Controlled motivation → Intention	0.09	0.02	[0.02, 0.12]
Attitude → FIB	0.01	0.36	[−0.02, 0.05]
Subjective norm → FIB	0.07	0.01	[0.01, 0.15]
PBC → FIB	0.10	0.20	[−0.05, 0.23]
Autonomous motivation → FIB	0.13	0.003	[0.04, 0.22]
Controlled motivation → FIB	0.02	0.23	[−0.01, 0.05]

*β* = standardized parameter estimate, 95% confidence interval (CI) = 95% confidence interval, PBC = perceived behavioral control, theory of planned behavior (TPB) constructs = attitude, subjective norm, and PBC, FIB = fluid intake behavior.

## Supplementary Materials

The following is available online at http://www.mdpi.com/2072-6643/10/5/646/s1. Table S1: Scale items for constructs of the integrated model of pre-exercise fluid intake.
