# Supplementary material for: Predicting Athletes’ Pre-Exercise Fluid Intake: A Theoretical Integration Approach
Source: Nutrients. 2018 May 21;10(5):646. doi: 10.3390/nu10050646 (PMC5986525; doi:10.3390/nu10050646)
Supplement: Supplementary file 1 [file nutrients-10-00646-s001.pdf]

**Table S1.** Scale items for constructs of the integrated model of pre-exercise fluid intake

| Variable | Item | Scale |
|---|---|---|
| Autonomous motivation | 我会在运动前补充充足的水分，… [I want to intake sufficient fluid before exercise, …]<br>1. …因为我想对自己的健康负责 […because I want to take responsibility for my own health]<br>2. …因为我相信为了我的运动表现，这是应该做的事 […because it is the best thing for my sports performance]<br>3. …因为这有助于我保持自己的运动表现 […because it is very helpful for maintaining my sports performance]<br>4. …因为这是一个我很想做的重要决定 […because it is an important choice I really want to make]<br>5. …因为这和我内心深处的想法一致 […because it is consistent with my life goals]<br>6. …因为尽可能的保持身体水分平衡是很重要的 […because it is very important for being as well-hydrated as possible] | 1 = "not at all true", 7 = "very true" |
| Controlled motivation | 我会在运动前补充充足的水分，… [I want to intake sufficient fluid before exercise, …]<br>1. …因为如果我不这样做便会感到羞愧 […because I would feel ashamed of myself if I did not]<br>2. …因为如果我不这样做，会令其他人不开心 […because others would be upset with me if I did not]<br>3. …因为我不这样做，我会觉得不好 […because I would feel bad about myself if I did not]<br>4. …因为按照别人的指示做，比自己想如何去做容易 […because it is easier to do what I am told than think about it]<br>5. …因为我需要得到别人对我的认可 […because I want others to approve of me]<br>6. …因为我想要其它人看到我能做到 […because I want others to see I can do it] | 1 = "not at all true", 7 = "very true" |
| Attitude | 运动前补充充足的水分是… [Intake sufficient fluid before exercise is …]<br>1. 不重要的—重要的 [worthless—valuable]<br>2. 有害的—有益的 [harmful—beneficial]<br>3. 不愉快的—愉快的 [unpleasant—pleasant]<br>4. 不享受的—享受的 [unenjoyable—enjoyable]<br>5. 不好的—好的 [bad—good] | 1 = "worthless", 7 = "valuable"<br>1 = "harmful", 7 = "beneficial"<br>1= "unpleasant", 7 = "pleasant"<br>1 = "unenjoyable", 7 = "enjoyable"<br>1 = "bad", 7 = "good" |
| Subjective norm | 1. 很多对我来讲很重要的人认为我需要在未来一个月里的运动前补充充足的水分 [Most people who are important to me think that I should consume sufficient fluid before exercise in the forthcoming month]<br>2. 在未来一个月里的运动前补充充足的水分是大家认为理所当然的事 [It is expected of me that I consume sufficient | 1 = "strongly disagree", 7 = "strongly agree" |

| | | |
|---|---|---|
| | fluid before exercise in the forthcoming month]<br>3. 在生命中我重视他们意见的人都会同意在未来一个月里的运动前补充充足的水分 [The people in my life whose opinions I value would approve of me consuming sufficient fluid before exercise in the forthcoming month] | |
| Perceived behavioral control | 1. 对我来讲在未来一个月里的运动前补充充足的水分是可能的 [It is possible for me to consume sufficient fluid before exercise in the forthcoming month]<br>2. 对我来讲在未来一个月里的运动前补充充足的水分是容易的事 [It is easy for me to consume sufficient fluid before exercise in the forthcoming month]<br>3. 只要我愿意，便可以在未来一个月里的运动前补充充足的水分 [If I want to I could consume sufficient fluid before exercise in the forthcoming month]<br>4. 我完全能控制在未来一个月里的运动前补充充足的水分 [I have complete control over consuming sufficient fluid before exercise in the forthcoming month]<br>5. 在未来一个月里的运动前补充充足的水分基本取决于我自己意愿 [It is mostly up to me whether or not I consume sufficient fluid before exercise in the forthcoming month] | 1 = "strongly disagree", 7 = "strongly agree" |
| Intention | 1. 我打算在未来一个月里的运动前补充充足的水分 [I intend to consume sufficient fluid before exercise in the forthcoming month]<br>2. 我将会试图努力在未来一个月里的运动前补充充足的水分 [I will try to put great effort into consuming sufficient fluid before exercise in the forthcoming month]<br>3. 我计划在未来一个月里的运动前补充充足的水分 [I plan to consume sufficient fluid before exercise in the forthcoming month] | 1 = "strongly disagree", 7 = "strongly agree" |
| Pre-exercise fluid intake behavior | 1. 我会观察自己早晨起床后尿液的颜色 [I will observe my urine color after I wake up in the morning]<br>2. 我会观察自己运动前三到四小时的尿液颜色 [I will observe my urine color three to four hours prior to exercise]<br>3. 我会根据气温来调整我运动前的饮水量 [I will adjust the volume of fluid intake before exercise based on the temperature]<br>4. 我会根据自己体重的变化来调整我运动前的饮水量 [I will adjust the volume of fluid intake before exercise based on my weight change] | 1 = "almost never", 5 = "almost always" |
